# Ten-Fraction Stereotactic Radiosurgery With Different Gross Tumor Doses and Inhomogeneities for Brain Metastasis of >10 cc: Treatment Responses Suggesting Suitable Biological Effective Dose Formula for Single and 10 Fractions

**DOI:** 10.7759/cureus.34636

**Published:** 2023-02-04

**Authors:** Kazuhiro Ohtakara, Kiyo Nakabayashi, Kojiro Suzuki

**Affiliations:** 1 Department of Radiation Oncology, Kainan Hospital Aichi Prefectural Welfare Federation of Agricultural Cooperatives, Yatomi, JPN; 2 Department of Radiology, Aichi Medical University, Nagakute, JPN; 3 Department of Neurosurgery and Neuroendovascular Therapy, Yokkaichi Municipal Hospital, Yokkaichi, JPN

**Keywords:** stereotactic radiosurgery, linear-quadratic-linear model, linear-quadratic-cubic model, linear-quadratic model, large tumor, fractionation, dynamic conformal arcs, brain metastasis, biological effective dose, alpha/beta ratio

## Abstract

Stereotactic radiosurgery (SRS) with >5 fractions (fr) has been increasingly adopted to improve local control and safety for brain metastases (BM) of >10 cm^3^, given the limited brain tolerance of SRS with ≤5 fr. However, the optimal indication and treatment design, including the prescribed dose and distribution for 10 fr SRS, remains uncertain. A single fr of 24 Gy provides approximately 95% of the one-year local tumor control probability. The potential SRS doses in 10 fr that is clinically equivalent to a single fr of 24 Gy regarding anti-tumor effect range from 48.4 to 81.6 Gy as biological effective doses (BED) as a function of the BED model formulas along with the alpha/beta ratios. The most appropriate BED formula in conjunction with an alpha/beta ratio to estimate similar anti-BM effects for single and 10 fr remains controversial.

Herein, we describe four cases of symptomatic radiation-naïve BM >10 cm^3^ (range, 11 to 26 cm^3^), treated with 10 fr SRS with a standard prescribed dose of 42 Gy, for which modified dynamic conformal arcs were used with forward planning to improve dose conformity. In the first two cases with gross tumor volumes (GTV) of 15.3 and 10.9 cm^3^, 42 Gy was prescribed to 70%-80% isodose, normalized to 100% at the isocenter, which encompasses the boundary of the planning target volume: GTV + isotropic 1 mm margin. The tumor responses were initially marked regression followed by regrowth within three months in case 1 and no shrinkage with subsequent progression within three months in case 2. In the remaining two cases with larger GTVs of 19.1 and 26.2 cm^3^, the GTV boundary and 2-3 mm margin-added object volume was covered by 80% and 56% isodoses with 53 Gy and 37 Gy, respectively, to further increase the marginal and internal doses of GTV and to ensure moderate dose spillage outside the GTV, while >1-1.5 mm outside the GTV was covered by 42 Gy with 63% isodose. According to the BED based on the linear-quadratic (LQ) model with an alpha/beta ratio of 10 (BED_10_), 53 Gy corresponds to approximately 81 Gy in BED_10_ and 24 Gy in a single fr. Excellent initial maximum tumor response and subsequently sustained tumor regression (STR) were achieved in both cases. Subsequently, enlarging nodules that could not exclude the possibility of tumor regrowth were disclosed within two years, while late adverse radiation effects remained moderate.

These dose-effect relationships suggest that a GTV marginal dose of ≥53 Gy with ≤80% isodose would be preferred to effect ≥1-year STR and that further dose escalation of both marginal and internal GTV may be necessary to achieve ≥2-year STR, while GTV of >25 cm^3^ may be unsuitable for 10 fr SRS in terms of long-term brain tolerance. Among LQ, LQ-cubic, and LQ-linear model formulas and alpha/beta ratios of 10-20, BED_10_ may be clinically most suitable to estimate a 10 fr SRS dose that provides anti-BM efficacy similar to that for a single fr.

## Introduction

Surgical en bloc resection followed by postoperative irradiation, if necessary, has been positioned as the mainstay of treatment for symptomatic large brain metastases (BM) of >10 cm^3^, especially those that manifest severe mass effects. However, craniotomy entails several inherent detriments, including invasiveness, discontinuation of anti-cancer medications, and the risk of triggering tumor cell seeding [1,2]. Therefore, multi-fraction (fr) stereotactic radiosurgery (SRS) has been keenly anticipated as a far less invasive alternative to open surgery [2,3]. However, local control (LC) of large BM of >10 cm^3^ with ≤5 fr SRS remains unsatisfactory, as prescribed marginal doses are frequently reduced for larger BM considering the volume effect on brain tolerance [2-5]. The persistence of adopting limited- and fixed-dose fractions and the compromise of the prescribed dose likely led to incomplete tumor necrosis and/or clinically overt brain injury, therefore impeding the probability of excellent LC [3,4]. Therefore, SRS with >5 fr, exempli gratia 10 fr, has been increasingly adopted to improve LC and safety for BM of >10 cm^3^ without the urgent need to alleviate the mass effect, considering the recent recognition of much less limited brain tolerance for ≤5 fr than previously assumed [4-8]. However, the optimal indication, prescribed dose, and dose distribution for 10 fr SRS remain unestablished. These uncertainties include target volume definition, prescribed isodose, coverage, target dose inhomogeneity, and brain tolerance, in which substantial inter-institutional differences and variabilities remain [6-8]. A single fr of 24 Gy provides approximately 95% of the one-year local tumor control probability (TCP). Meanwhile, 3-5 fr SRS with 27-35 Gy for BM of 21-40 mm in diameter yields a lower 1-year LC of approximately 80% [3]. The 10 fr SRS dose that provides an anti-tumor effect clinically equivalent to that of 24 Gy in 1 fr or yields a 1-year local TCP of ≥95% for BM of >10 cm^3^ remains uncertain [6-8]. The most appropriate biological effective dose (BED) formula, along with an alpha/beta ratio that estimates similar anti-BM efficacy for SRS doses in 10 fr and 1 fr, has continued to be controversial [8,9].

Herein, we describe four cases of symptomatic radiation-naïve BM of >10 cm^3^, ranging from 11 to 26 cm^3^, treated with 10 fr SRS with the standard prescribed marginal dose of 42 Gy during 10 months, for which a micro-multileaf collimator (mMLC)-based modified dynamic conformal arc (mDCA) was used with forward planning [10]. In response to short-term inadequate efficacies in the first two cases of BM <16 cm^3^, both marginal and internal dose escalation of the gross tumor volume (GTV) and a sufficient dose spillage margin outside of the GTV were adopted for the remaining two cases with a larger BM (>19 cm^3^), resulting in >1-year sustained tumor regression (STR) along with moderate adverse radiation effect (ARE). Based on the dose-effect relationships, the optimal dose and distribution in 10 fr to achieve superior tumor response and its long-term sustainment will be discussed, specifically regarding the suitable GTV marginal dose and dose gradient inside and outside the GTV boundary. Additionally, among various BED model formulas and alpha/beta ratios, it is also considered that the most appropriate BED model formula in conjunction with an alpha/beta ratio that estimates the SRS dose in 10 fr provides an anti-BM effect similar to that for an SRS dose in single fr.

Part of this study was previously presented at the 30^th^ Annual Meeting of the Japanese Society for Stereotactic Radiosurgery, held online on June 11 to July 8, 2021.

## Case presentation

**Figure 1:**
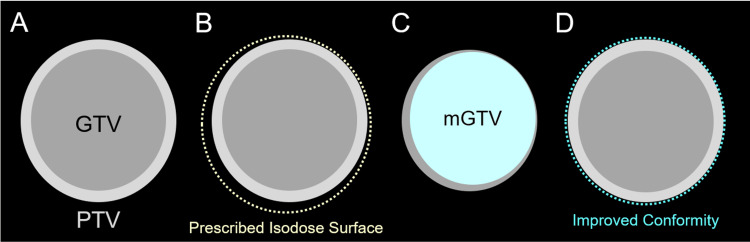
Forward planning method for modified dynamic conformal arcs. The images show target volume definition consisting of gross tumor volume (GTV) and planning target volume (PTV) (A); PTV covering by prescribed isodose surface (IDS) in general dynamic conformal arcs (DCA) (B); modified GTV (mGTV) compared to the original GTV (C); and PTV covering by prescribed IDS in modified DCA (mDCA) (D). (B) In this case, when the original GTV is directly used as an object volume for leaf adaptation, the dose conformity of the PTV with the prescribed IDS (dashed line) has room for improvement, that is, over-coverage. (C) To improve the dose conformity, a copy of the original GTV is used to generate a surrogate object for leaf adaptation to be deformed in a desired shape, namely, the mGTV, and then modified according to the over- and/or under-coverage of the PTV with the initial prescribed IDS. (D) When an appropriately modified mGTV is used for leaf adaptation, the dose conformity of the PTV with the prescribed IDS is improved.

Four patients with symptomatic radiation-naïve BM >10 cm^3^ were treated within 15 months after the start of SRS using a newly installed linear accelerator. All SRS was performed with mDCA using Novalis Truebeam STx® (Palo Alto, CA: Varian Medical Systems) with a flattening-filter-free mode of a 6 megavoltage (MV) X-ray beam, which offers a dose rate of up to 1400 MU/min. This platform has an integrated high-definition mMLC, HD120, with a central leaf width of 2.5-mm. The dosimetric characteristics of usual DCA with HD120 and the differences between 2.5-mm and 3-mm leaf width have previously been described [10-12]. The treatment planning system was the iPlan Dose (Munich, Germany: Brainlab AG), in the then-version, for which forward planning was only available for DCA [11,12]. The arc arrangement consisted of one coplanar arc and two non-coplanar arcs allocated to divide the cranial hemisphere evenly. The method of mDCA is illustrated in Figure 1, and the other utility has been described previously [13].

GTV was defined as enhancing lesions, as the enhancing lesions were equal to or slightly larger than the visible mass on T2-weighted images (T2-WI) without prominent exudation of contrast medium in all cases [14]. The clinical characteristics and planning parameters of the four cases are summarized in Table 1.

**Table 1:** Case descriptions and planning parameters of 10-fraction stereotactic radiosurgery. Primary: primary organ; Int.: The interval between the acquisition of magnetic resonance images for planning and the initiation of stereotactic radiosurgery; GTV: gross tumor volume; Max.: maximum; PTV: planning target volume; TTP: time to tumor progression; BED_10_: biological effective dose based on the linear-quadratic model with an alpha/beta ratio of 10; % isodose: reference isodose normalized to 100% at the isocenter; SCC: squamous cell carcinoma; LCC: large cell carcinoma; Adeno: adenocarcinoma; IDC: invasive ductal carcinoma Absolute doses of 66.3 Gy, 53 Gy, 42 Gy, and 37 Gy in 10 fractions correspond approximately to 110.3 Gy, 81.1 Gy, 59.6 Gy, and 50.7 Gy for BED_10_ and 28.6 Gy, 23.9 Gy, 19.9 Gy, and 18.1 Gy for absolute dose in single fraction estimated by BED_10_, respectively.

Case	Primary	Int. (days)	GTV	GTV Boundary				Isocenter		PTV Boundary			TTP (month)
			(Max. diameter)										
	(Pathology)												
				Absolute dose	Coverage	BED_10_	% isodose	Absolute dose	BED_10_	Margin	Absolute dose	Coverage	
1	Esophagus	4	15.3 cm^3^	45.7 Gy	99%	66.6 Gy	87.0%	52.5 Gy	80.1 Gy	2 mm	39.4 Gy	98%	2.5-4.5
	(SCC)												
			(38 mm)										
2	Lung	6	10.9 cm^3^	49.0 Gy	99%	73.0 Gy	81.7%	60.0 Gy	96.0 Gy	2 mm	34.8 Gy	98%	2.5
	(LCC)												
			(33 mm)										
3	Lung	6	19.1 cm^3^	53.0 Gy	97%	81.1 Gy	80.0%	66.3 Gy	110.3 Gy	2 mm	37.0 Gy	98%	(18.0-20.7)
	(Adeno)												
			(38 mm)										
4	Breast	10	26.2 cm^3^	55.0 Gy	100%	85.3 Gy	83.0%	66.3 Gy	110.3 Gy	(2 mm)	(42.5 Gy)	(98%)	(17.0)
	(IDC)												
			(49 mm)							3 mm	37.0 Gy	99%	

Differences in BED to estimate the anti-tumor effect and the corresponding physical doses in 10 fr equivalent to a single dose of 24 Gy are tabulated in Table 2 as a function of the BED model formulas and the values of the alpha/beta ratio [9].

**Table 2:** Difference and variation of biologically effective doses and corresponding absolute doses in 10 fractions equivalent to a single fraction of 24 Gy as a function of the model formula and alpha/beta ratio. LQ: linear-quadratic; LQC: linear-quadratic-cubic; LQL: linear-quadratic-linear; BED: biological effective dose; fr: fraction

Model Formula	LQ	LQ	LQ	LQC	LQC	LQL
Alpha/beta ratio	10	12	20	10	12	10
BED	81.6 Gy	72.0 Gy	52.8 Gy	56.0 Gy	50.7 Gy	48.4 Gy
Absolute dose (10 fr)	53.2 Gy	50.6 Gy	43.4 Gy	40.7 Gy	39.0 Gy	36.9 Gy

Notably, the corresponding absolute doses in 10 fr range from 36.9 to 53.2 Gy, and the BED ranges from 48.4 to 81.6 Gy.

To review and compare serial magnetic resonance images before and after SRS as precisely and objectively as possible, the image datasets, including contrast-enhanced (CE) T1-weighted images (T1-WI) and T2-WI for each case, were co-registered on the dedicated workstation MIM Maestro^TM^ (Cleveland, OH: MIM Software), and then fused to coincide with each other through the cranium [8]. Although slight blurring was observed in some images as a result of image co-registration, the enlargement rate and coordinates were aligned to ensure the accuracy of T1/T2 matching, in which a visible mass was compared between CE-T1-WI and T2-WI to determine whether tumor regrowth or ARE was dominant for an enlarging lesion [8,14].

Case 1

**Figure 2:**
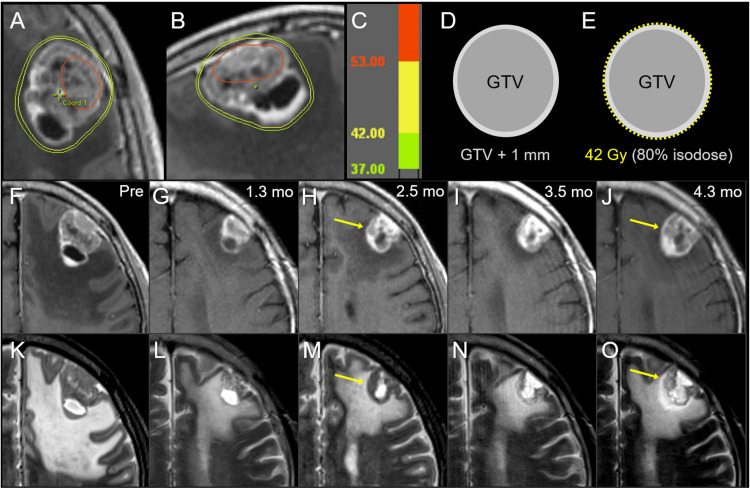
Case 1: Dose distribution, planning design, and images before and after 10-fraction stereotactic radiosurgery. The images show (A, B) dose distributions (A: axial view; B: sagittal view); corresponding isodoses (C); schema of the target definition, including GTV and PTV: GTV + isotropic 1 mm (D); schema of treatment planning (E); contrast-enhanced (CE) T1-weighted images (T1-WI) (F-J); and T2-weighted images (T2-WI) (K-O); 4 days before the initiation of stereotactic radiosurgery (SRS) (Pre) (F, K); at 1.3 months (mo) after the initiation of SRS (G, L); at 2.5 months (H, M); at 3.5 months (I, N); and 4.3 months (J, O). (A) The irradiated tumor volume of 53 Gy (red) is extremely limited. (E) A dose of 42 Gy is prescribed for 80% IDS, normalized to 100% at the isocenter, encompassing the PTV boundary. (F-O) On T1/T2 matching, tumor regrowth is observed once at 2.5 months (arrows in H, M) and again at 4.3 months (arrows in J, O), while tumor regression is markedly observed at 1.3 months and slightly at 3.5 months. GTV: gross tumor volume; PTV: planning target volume; IDS: isodose surface

A 61-year-old male presented impaired consciousness and motor dysphasia and was clinically diagnosed with BM in the left frontal lobe two years after the diagnosis of esophageal squamous cell carcinoma (Figure 2).

The patient’s past 23-month anti-cancer treatment included induction chemotherapy (CTx) with cisplatin and 5-fluorouracil (FP); definitive surgery; salvage medication with the first line of FP, the second line of nivolumab, and the third​​​​​​​ line of docetaxel (DTX) for recurrence in the mediastinal lymph node (LN) 9.5 months after surgery; and palliative radiotherapy (RT) for thoracic spine metastases. Karnofsky's performance status (KPS) before SRS was 50%, and other extracranial active lesions at the time of BM diagnosis were multiple lung and liver metastases. SRS was initiated 21 days after the introduction of DTX. The patient’s neurological condition gradually and markedly improved during and after SRS. Anti-cancer treatments after SRS were CTx with paclitaxel (PTX) 2.2 months after the initiation of SRS, palliative RT for supraclavicular LN metastasis and chest wall invasion from pleural dissemination, and additional SRS for newly disclosed limited BM at 3.0 and 5.3 months.

The tumor response after SRS is described in detail in Figure 2. The maximum tumor response resulted in a partial response at 1.3 months, suggesting residual viable tissue, with subsequent smoldering and regrowth within three months, which was curbed by CTx to some extent. The patient died 5.5 months after SRS, mainly due to the progression of pleural dissemination.

Case 2

**Figure 3:**
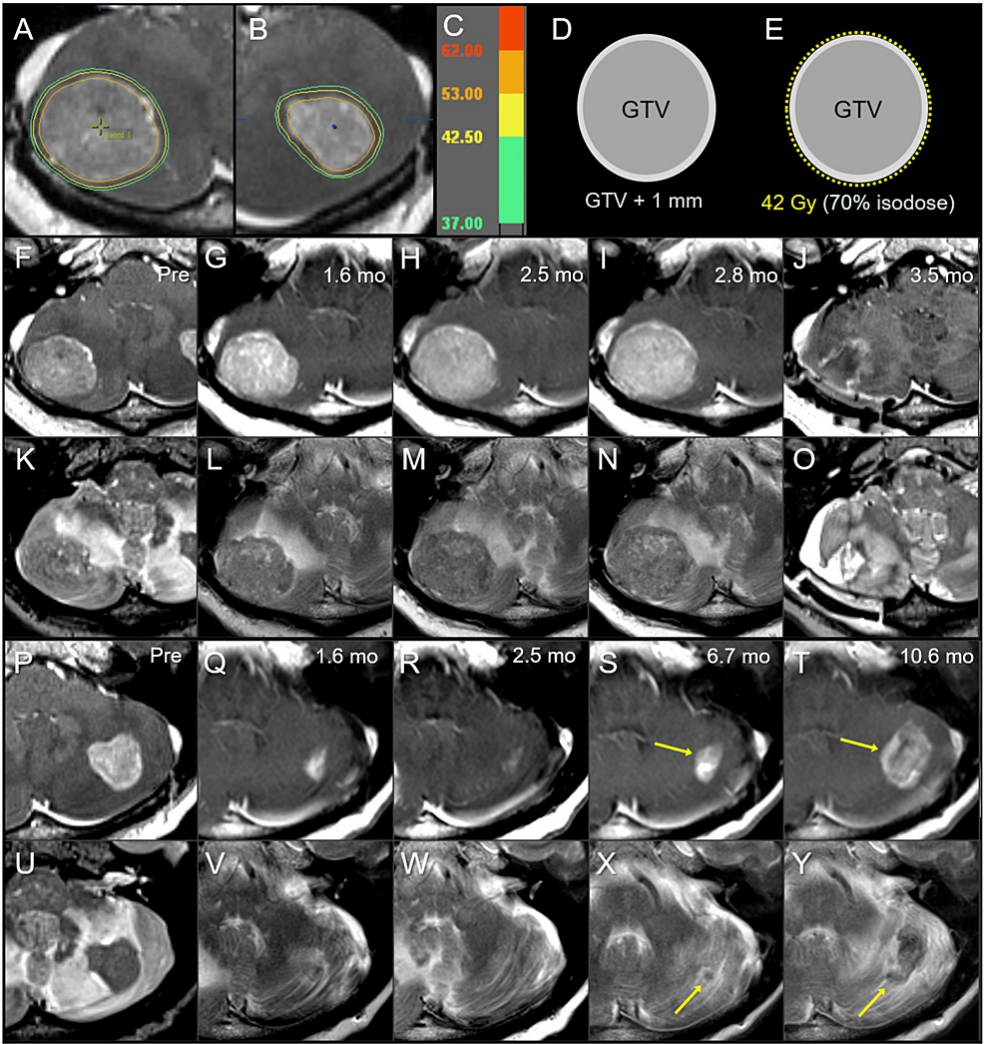
Case 2: Dose distribution, planning design, and images before and after 10-fraction stereotactic radiosurgery. The images show (A, B) axial views of dose distributions in the right (A) and left (B) cerebellar lesions; corresponding isodoses (C); schema of the GTV and PTV: GTV + 1 mm (D); schema of treatment planning (E); the right cerebellar lesion (F-O); the left cerebellar lesion (P-Y); CE-T1-WI (F-J, P-T); T2-WI (K-O, U-Y); six days before the initiation of SRS (Pre) (F, K, P, U); at 1.6 months (mo) after the initiation of SRS (G, L, Q, V); at 2.5 months (H, M, R, W); at 2.8 months (I, N); at 3.5 months (J, O); at 6.7 months (S, X); and 10.6 months (T, Y). (A, B) The substantial volumes of both GTVs are covered by 53 Gy (orange). (E) 42 Gy is prescribed to 70% IDS covering the PTV. (F-O) The right cerebellar lesion showed no regression at 1.6 months (G, L), with subsequent enlargement of up to 39 mm in maximum diameter at 2.8 months (I, N). (P-Y) The left cerebellar lesion achieved a nearly complete remission at 2.5 months (Q, R, V, W); however, enlargement of both the enhancing lesion and iso-intensity to a low-intensity nodule on T2-WI are observed at 6.7-10.6 months (S, T, X, Y). GTV: gross tumor volume; PTV: planning target volume; CE: contrast-enhanced; T1-WI: T1-weighted image; T2-WI: T2-weighted image; SRS: stereotactic radiosurgery; IDS: isodose surface

A 50-year-old male presented headache and unsteadiness and was diagnosed with BM in the bilateral cerebellar hemispheres two years after the diagnosis of locally advanced large cell carcinoma of the lung (Figure 3).

The patient’s past 22.4-month anti-cancer treatment included concurrent chemoradiotherapy (CCRT) with weekly carboplatin (CBDCA) and PTX followed by consolidative CTx, the second​​​​​​​ line of DTX, the third line of CBDCA and pemetrexed (PEM), and the fourth line of nivolumab. The KPS before SRS was 80%, and the extracranial active disease at the time of BM diagnosis included pararenal retroperitoneal metastasis.

SRS was started 28 days after the 23^rd^ course of nivolumab, and the fifth ​​​​​​​line of vinorelbine was administered 1.5 months after the initiation of SRS. However, the patient presented with disturbed consciousness attributed to obvious tumor progression in the right cerebellar tumor 2.3 months after the initiation of SRS and underwent a lesionectomy three months after the initiation of SRS. As the predominance of viable tissue was verified by pathological examination, 10 fr of SRS was added to the resection cavity 24 days after craniotomy. As a reference, the treatment response for a left cerebellar BM of 5.0 cm^3^ with a maximum diameter of 27 mm is shown in Figure 3 (P-Y), strongly suggesting an insufficient tumor dose. Other anti-cancer treatments after SRS included oral administration of S-1, limited to eight days due to adverse reactions, whole brain RT (WBRT) with 30 Gy in 10 fr for newly developed multiple BM 10.6 months after the initial SRS and palliative RT for skin metastasis. The patient died 13.8 months after SRS, mainly due to the progression of extracranial disease.

Case 3

**Figure 4:**
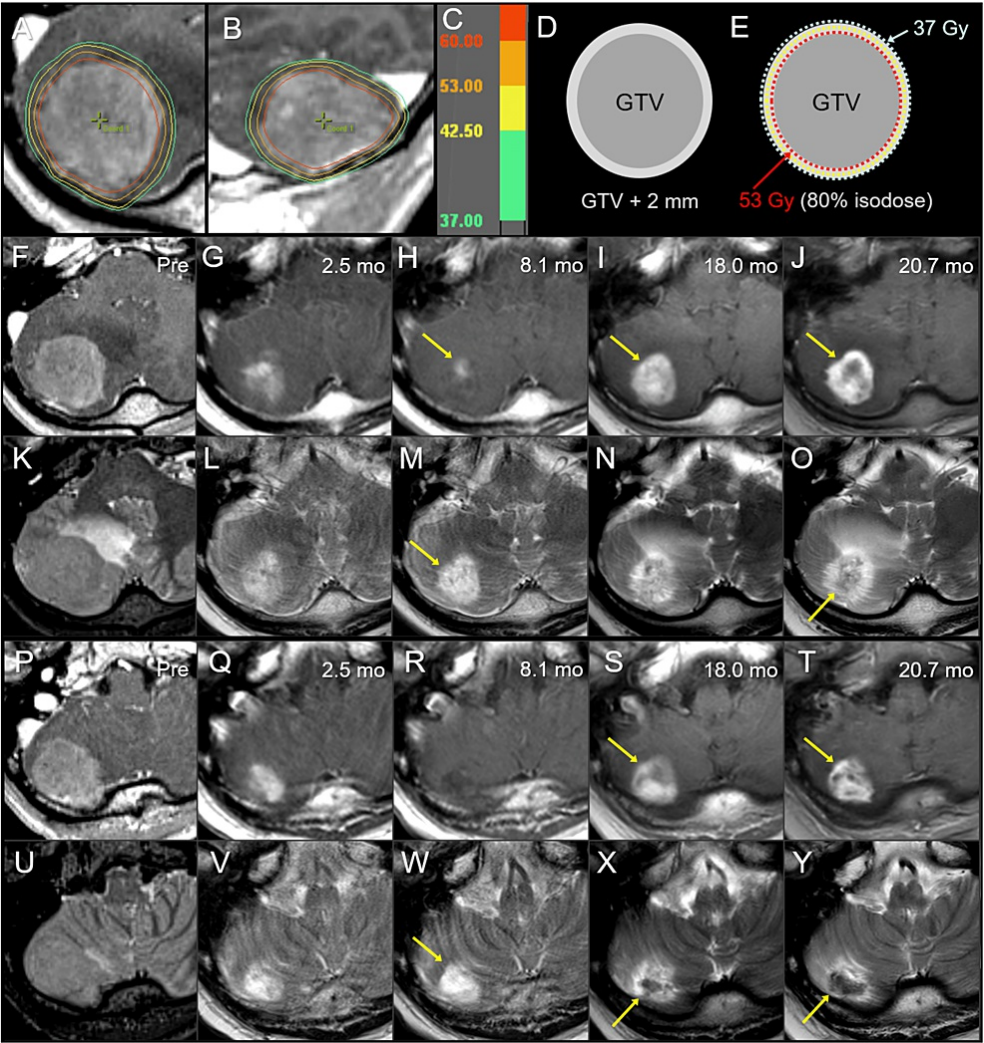
Case 3: Dose distribution, planning design, and images before and after 10-fraction stereotactic radiosurgery. The images show (A, B) dose distributions (A: axial view; B: sagittal view); corresponding isodoses (C); schema of GTV and object volume for dose evaluation: GTV + 2 mm (D); schema of treatment planning (E); axial views at the plane of initial GTV center (F-O); axial views at the more caudal plane (P-Y); CE-T1-WI (F-J, P-T); T2-WI (K-O, U-Y); six days before the initiation of SRS (Pre) (F, K, P, U); at 2.5 months (mo) after the initiation of SRS (G, L, Q, V); at 8.1 months (H, M, R, W); at 18.0 months (I, N, S, X); and at 20.7 months (J, O, T, Y). (A, B) The D_66%_ of the GTV is 62 Gy (red), in which D_X%_ indicates the dose irradiated to at least X% of the volume. (E) 53 Gy is prescribed to 80% IDS encompassing the GTV boundary, and the GTV + 2 mm boundary is simultaneously covered by 37 Gy. (G, L, Q, V) At 2.5 months, marked tumor regression is observed in which the iso-intensity mass in T2-WI almost disappeared, leaving a slight enhancement. (H, M, R, W) Subsequently, a marked discrepancy, i.e., T1/T2 mismatch, between the slightly enhancing lesion (arrow in H) and high-intensity tumor necrosis (arrows in M, W) is observed at 8.1 months. (I, N, S, X) At 18.0 months, enlargement of the enhancing lesion (arrows in I, S) up to the high-intensity lesion boundary on T2-WI and a low-intensity nodule on T2-WI (arrow in X) appeared. (J, O, T, Y) At 20.7 months, the low-intensity nodule (arrow in Y) enlarged, and an iso-intensity irregular mass (arrow in O) became noticeable, while the enhancing lesion (arrows in I, S), suggestive of the radiation effect, did not show significant enlargement (arrows in J, T). GTV: gross tumor volume; CE: contrast-enhanced; T1-WI: T1-weighted image; T2-WI: T2-weighted image; SRS: stereotactic radiosurgery; IDS: isodose surface

A 51-year-old male presented intermittent headache and was diagnosed with BM in the right cerebellar hemisphere without hydrocephalus 21.6 months after the diagnosis of locally advanced lung adenocarcinoma harboring an epidermal growth factor receptor gene mutation (Figure 4).

The patient’s past 19.6-month anti-cancer treatment included a first​​​​​​​ line of osimertinib, a second​​​​​​​ line with CBDCA + PEM, CCRT, a third​​​​​​​ line of nab-PTX, and a fourth​​​​​​​ line of S-1. The KPS before SRS was 80%, and the extracranial active disease at the time of BM diagnosis included intrathoracic progression and pleural dissemination. SRS was started 22 days after S-1 administration, and the patient’s neurological symptoms gradually improved and finally resolved during and after SRS. Oral administration of S-1 resumed 23 days after completion of SRS and finally discontinued 19.8 months after SRS initiation. The initial and maximum tumor responses and subsequent courses are described in detail in Figure 4. In summary, LC was achieved, and late ARE was moderate without the need for unplanned medication, while an enlarging nodule that could not exclude the possibility of tumor regrowth was observed at the last follow-up image. The patient died 30 months after SRS mainly because of the progression of intrathoracic disease and bacterial pneumonia.

Case 4

**Figure 5:**
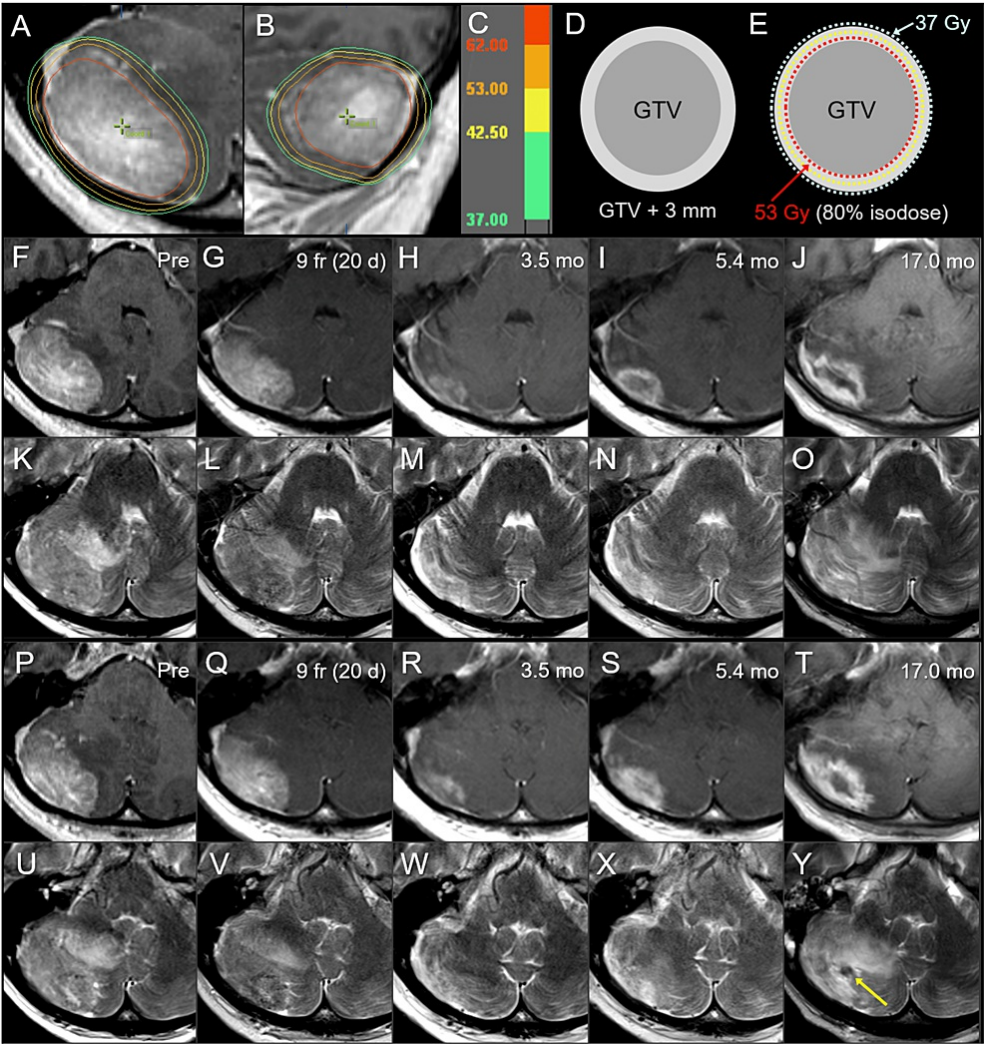
Case 4: Dose distribution, planning design, and images before and after 10-fraction stereotactic radiosurgery. The images show (A, B) dose distributions (A: axial view; B: sagittal view); corresponding isodoses (C); schema of GTV and object volume for dose evaluation: GTV + 3 mm (D); schema of treatment planning (E); axial views at the plane of initial GTV center (F-O); axial views at the more caudal plane (P-Y); CE-T1-WI (F-J, P-T); T2-WI (K-O, U-Y); 10 days before the initiation of SRS (Pre) (F, K, P, U); at nine fractions (fr) and also 20 days (d) after pre-image acquisition (G, L, Q, V); at 3.5 months (mo) after the initiation of SRS (H, M, R, W); at 5.4 months (I, N, S, X); and at 17.0 months (J, O, T, Y). (A, B) The D_85%_ of GTV is 62 Gy (red); (E) 53 Gy is prescribed for 80% IDS that fully encompasses the GTV boundary, and the GTV + 3 mm boundary is simultaneously covered by 37 Gy, given the presumed brain invasion and the 10-day interval between image acquisition and SRS initiation. (G, L, Q, V) Slight tumor regression is observed at 9 fr. (G, L, Q, V) A marked decrease in the enhancing lesion and a visible mass on T2-WI are observed at 3.5 months. (I, N, S, X) The increase in enhancement without enlarging the nodule on T2-WI suggested a radiation effect at 5.4 months. (J, O, T, Y) Further enlargement of the enhancing lesion, a low-intensity nodule on T2-WI (arrow in Y), and aggravation of perilesional edema are observed at 17.0 months. GTV: gross tumor volume; CE: contrast-enhanced; T1-WI: T1-weighted image; T2-WI: T2-weighted image; SRS: stereotactic radiosurgery; IDS: isodose surface

A 51-year-old female with a history of neurofibromatosis type 1 presented headache and gait unsteadiness and was diagnosed with BM in the right cerebellar hemisphere without hydrocephalus 21 months after the diagnosis of left-sided breast cancer harboring the HER2-positive luminal subtype (Figure 5).

The patient’s past 20.5-month anti-cancer treatment included neoadjuvant CTx, mastectomy without postoperative RT, trastuzumab, and tamoxifen. KPS before SRS was 80%, and extracranial active disease at the time of BM diagnosis included multiple bone metastases. The patient’s neurological symptoms gradually improved and resolved during and after SRS. Lapatinib and capecitabine were administered 20 days after the completion of the SRS, and multiple liver metastases were detected seven months after SRS.

The initial and maximum tumor responses and subsequent courses are described in detail in Figure 5. At 17.0 months, enlargement of the enhancing lesion without a corresponding mass on T2-WI, viz. T1/T2 mismatch was observed along with aggravation of perilesional edema, suggesting that brain radionecrosis was predominant, while a small enlarging nodule that could not exclude the possibility of tumor regrowth was observed. At 20.5 months, the patient’s neurological condition was stable under steroid administration, and was transferred to another hospital for subsequent care.

## Discussion

The GTV boundary with coverage of usually D_≥98%_, the dose irradiated to at least 98% of the GTV, has been a foundation and standard for dose prescription since the dawn of SRS, while the transition from dose prescription to the margin-added planning target volume (PTV) boundary has rendered the GTV marginal dose varied and vague [3,6,8,9,15]. Recent studies suggest that a higher proportion of GTV receiving ≥30-32 Gy in 1 fr, that is, internal dose escalation, is likely associated with superior TCP and/or tumor response [9]. Furthermore, the incidence and depth of microscopic tumor infiltration into the surrounding brain vary and tend to increase as a function of increased GTV and/or histopathology [16]. Therefore, complete tumor eradication, including brain invasion, is essential to achieve superior long-term tumor control. A dose fall-off outside the GTV boundary that is too steep can impede the control of brain invasion [8,16]. Consequently, the degree of dose gradient inside and outside the GTV boundary, as well as the GTV marginal dose, affects both tumor response and ARE. The optimal dose distribution for BM would be profoundly different from that for pathologically clearly demarcated benign tumors or vascular malformations. Considering the above background, the marginal doses of both GTV and 2-3 mm margin-added object volume were reviewed in addition to the original dose prescribed in the presented cases.

A GTV marginal dose of ≤49 Gy in cases 1 and 2 resulted in LC failure within three months, while that of ≥53 Gy in cases 3 and 4 led to >1-year STR. In case 1, the patient’s neurological symptoms improved markedly without subsequent deterioration due to limited survival despite early tumor progression. In case 2, SRS was completely ineffective for the right-sided tumor, despite the substantial proportion of both GTV covered with ≥53 Gy, while the dose fall-off outside the GTV boundary was rather steep, as the GTV + 2 mm marginal dose was 35 Gy. Additionally, the left-sided tumor showed nearly complete remission within three months; however, subsequently showed obvious regrowth within one year. These results indicate that an initial superior tumor response within a few months does not necessarily guarantee the probability of long-term superior LC, that is, optimal plan design, and that a higher dose of GTV is required to improve tumor response and subsequent sustainability. Therefore, we applied modified dose distributions to cases 3 and 4 to ensure both marginal and internal dose escalation of GTV and moderate dose spillage or attenuation margin outside the GTV boundary by reference to a previous report suggesting that 2-10 fr SRS with a BED_10_ of ≥80 Gy provides better LC for BM from non-small cell lung cancer [17]. The SRS principle for Kogo et al. was that physical doses equivalent to BED_10_ of ≥80 Gy were prescribed to a 90% isodose encompassing the D_95%_ of PTV (GTV + 1-2 mm), that is, a rather homogeneous dose of GTV [17]. Additionally, to minimize the risk of brain injury and to further enhance tumor response, we applied 53 Gy, BED_10_ >80 Gy, with 80% isodose to the GTV boundary, and concurrently, the GTV + 2-3 mm margin was covered by 37 Gy. The BED_10_ of 37 Gy in 10 fr was 50.7 Gy which is not inferior to 40 Gy in 20 fr, with BED_10_ of 48 Gy being a steady dose of WBRT. Despite the much larger GTV for cases 3 and 4, these dose escalations resulted in >1-year STR, although the persistence and regrowth of viable tumors within two years could not be denied.

Taken together, a GTV marginal dose of ≥53 Gy would be necessary to effect ≥1-year STR, and a further dose escalation of both the marginal and internal GTV dose, that is, the GTV margin covered by >53 Gy with <80% isodose may be necessary to achieve ≥2-year STR. Furthermore, these dose-effect relationships suggest that the anti-tumor effect of ≥53 Gy in 10 fr would approximate that of a single fr of 24 Gy and that BED_10_ based on the linear-quadratic (LQ) model with an alpha/beta ratio of 10 would be most suitable for estimating similar anti-tumor effects of 1 fr and 10 fr SRS. Although it has been asserted that the applicability of BED_10_ is inaccurate and should not be applied to clinical SRS, especially with ≤5 fr, it is vital to determine whether BED_10_ suits clinical BM, not cultured cell lines, as the tumor environments of BM and the brain-tumor interface are likely substantially heterogeneous and can also be altered as a function of GTV size and/or the effect of anti-cancer medication compared to those of monocellular-derived cultured cell lines. Furthermore, the suitable indications and targets are intrinsically different for single and 10 fr, particularly with respect to GTV size, that is, tumor cell number and intra- and peri-tumoral environments.

Regarding safety, in case 4 with the GTV of 26.2 cm^3^, the ARE required continued administration of steroids, suggesting that GTV >25 cm^3^ may be unsuitable for 10 fr SRS in terms of brain tolerance. If the alpha/beta ratio for the late effect on the normal brain is assumed to be 2, the BED based on the LQ model with an alpha/beta ratio of 2, namely, BED_2_, for 37 Gy, 42 Gy, and 53 Gy in 10 fr are 105.5 Gy, 130.2 Gy, and 193.5 Gy, respectively. These doses of BED_2_ were almost equivalent to 53 Gy, 65 Gy, and 97 Gy in 2 Gy per fraction. By contrast, BED_2_ for 18 Gy, 20 Gy, and 24 Gy in 1 fr are 180 Gy, 220 Gy, and 312 Gy, respectively, and 53 Gy in 10 fr is equivalent to 18.7 Gy in 1 fr for BED_2_. Further investigation is needed to elucidate the dose-volume relationships in 10 fr regarding late brain injury.

This report has several limitations, and arguments based on these cases require further investigation. In 10 fr SRS, there are possibilities of tumor volume/configuration changes and/or deviation within the cranium, the standard for image guidance, during the period from planning image acquisition to treatment completion [18,19]. However, in the presented cases, the interval between image acquisition and the initiation of SRS ranged from 4 to 10 days due to the limited accessibility to MRI examination and the need for considerable preparation time [18]. Although sufficient GTV coverage with 53 Gy and MRI evaluation at 9 fr were considered for case 4 with a 10-day interval, the presence of GTV enlargement during the waiting period and/or target change/displacement during 10 fr SRS remained unconfirmed in all cases [19]. SRS should commence within three days after planning image acquisition to minimize tumor enlargement/displacement during the waiting period [20]. Furthermore, it is indisputable that the four cases had extremely limited experience and that the initial and subsequent diagnoses of BM were based on clinical-radiographical findings, not pathological verification, except for case 2.

The presented cases and arguments warrant further investigations to determine the optimum indication and planning design, including GTV marginal dose and dose gradient inside and outside the GTV boundary in 10 fr SRS.

## Conclusions

Ten-fr SRS with sufficient GTV dose and an appropriate dose gradient near the GTV boundary can be an alternative to open surgery for BM of >10 cm^3^ without requiring urgent decompression. GTV marginal dose of ≥53 Gy in 10 fr, corresponding to the BED_10_ of ≥80 Gy, with ≤80% isodose, would be necessary to provide ≥1-year STR, while GTV >25 cm^3^ may not be suitable for 10 fr SRS was given brain tolerance. BED_10_ based on the LQ model with an alpha/beta ratio of 10 may be clinically the most suitable for estimating a 10 fr SRS dose that provides a tumor response similar to that for a single dose, among LQ, LQ-cubic, and LQ-linear model formulas, along with alpha/beta ratios of 10-20.
